# Prevalence of preterm labor in Iran: A systematic review and meta-analysis

**Published:** 2015-12

**Authors:** Katayon Vakilian, Mehdi Ranjbaran, Mahboobeh Khorsandi, Naser Sharafkhani, Mahmoud Khodadost

**Affiliations:** 1 *Faculty of Nursing and Midwifery, Arak University of Medical Sciences, Arak, Iran.*; 2 *Department of Epidemiology, Faculty of Health, Arak University of Medical Sciences, Arak, Iran.*; 3 *Department of Health Education and Health Promotion, Faculty of Health, Arak University of Medical Sciences, Arak, Iran.*; 4 *Gastroenterology and Liver Research Center, Baqiyatallah University of Medical Sciences, Tehran, Iran.*; 5 *Department of Epidemiology and Biostatistics, School of Public Health, Iran University of Medical Sciences, Tehran, Iran.*

**Keywords:** *Preterm labor*, *Prevalence*, *Meta-analysis*, *Iran*

## Abstract

**Background:**

Preterm labor, which defines as live-birth delivery before 37 weeks of gestation is a main determinant of neonatal morbidity and mortality around the world.

**Objective:**

The aim of this study was to determine the prevalence of preterm labor in Iran by a meta-analysis study, to be as a final measure for policy makers in this field.

**Materials and Methods:**

In this meta-analysis, the databases of Thomson database (Web of Knowledge), PubMed/Medline, Science Direct, Scopus, Google Scholar, Iranmedex, Scientific Information Database (SID), Magiran, and Medlib were searched for articles in English and Persian language published between 1995 and 2014. Among the studies with regard to the inclusion and exclusion criteria, 14 studies (out of 1370 publications) were selected. Data were analyzed by using Stata software version 11. The heterogeneity of reported prevalence among studies was evaluated by the Chi-square based Q test and I2 statistics.

**Results:**

The results of Chi-square based on Q test and I2 statistics revealed severe heterogeneity (Q=2505.12, p-value < 0.001 and I2= 99.5%) and consequently, the random effect model was used for the meta-analysis. Based on the random effect model, the overall estimated prevalence of preterm in Iran was 9.2% (95% CI: 7.6 – 10.7).

**Conclusion:**

Present study summarized the results of previous studies and provided a comprehensive view about the preterm delivery in Iran. In order to achieve a more desirable level and its reduction in the coming years, identifying affecting factor and interventional and preventive actions seem necessary.

## Introduction

Preterm labor, which defines as live-birth delivery before 37 completed weeks of gestation, is a main determinant of neonatal morbidity and mortality around the world (1, 2). Gestational age less than 28 weeks is defined as extremely preterm, 28 to <32 weeks as very preterm and 32 to <37 weeks as moderate to late preterm. Prematurity is the leading cause of babies' deaths in the first 4 weeks of life and the second leading cause of death after pneumonia in children under 5 years of age (3).

Despite advancing knowledge about risk factors and mechanisms of preterm labor, preterm birth rates are increasing in almost all countries and the development of public health and medical interventions designed to reduce preterm birth (2, 4, 5). Annually about 15 million babies are born prematurely and one million children die from complications of premature birth (3).

Hyaline membrane disease, bronchopulmonary dysplasia, patent ductus arteriosus, necrotizing enterocolitis and intraventricular hemorrhage are short-term complications of preterm birth, and neurological disorders such as cerebral palsy, hydrocephalus, seizures, vision and hearing disorders, and airway disorders are its main long-term complications (6). It should be mentioned that, about three-quarters of the deaths due to preterm birth complications, can be reduced even without neonatal intensive care (3).

Preterm birth is a syndrome with a variety of causes, which classified into: spontaneous preterm birth and provider-initiated preterm birth (7, 8). Previous preterm birth, black race, periodontal disease, and low maternal body-mass index are risk factors of spontaneous preterm births (2, 9, 10). Based on some studies in Iran, low general health status, history of disease during pregnancy, family history of prematurity, previous preterm labor, history of previous neonatal death, periodontal disease, decreased amniotic fluid, multiple pregnancies, infertility, and cervical incompetence were identified as risk factors of preterm labor (11-13).

Considering the importance of preterm birth, the latest statistics in a country can help plan the programs and introduction of guidelines for its reduction and control. On the other hand, due to multiplicity of studies, the need for an accurate and reliable result in this area seems essential.

Meta-analysis by combining results from different studies can increase the sample size, statistical power and precision and provide an overall quantitative result (14, 15). The aim of this study was to determine the prevalence of preterm labor in Iran by a meta-analysis study, to be as a final measure for policy makers in this field.

## Materials and methods

This meta-analysis was conducted based on the relevant empirical literature about the prevalence of preterm delivery in Iran. The international databases, including Thomson (Web of Knowledge), PubMed/Medline, Science Direct, Scopus, Google Scholar, Iranmedex, Scientific Information Database (SID), Magiran, and Medlib were systematically searched for articles in English and Persian language published between 1995 and 2014.

The search was performed with all possible combinations of key words; Preterm labor, preterm birth, preterm delivery, and Iran. The search strategy was [“Preterm” OR “Preterm labor” OR “preterm birth” OR “Preterm delivery” AND “Iran”].

All English or Persian electronic letratures that studied the prevalence of preterm labor in each province of Iran were included. Studies in special populations such as employed women, studies in referral centers for high risk pregnancy, and duplicated articles were excluded.

The quality assessment of eligible papers has been followed independently by two researchers using the Strengthening the Reporting of Observational Studies in Epidemiology (STROBE) Statement (16), and probable disagreement between them resolved through discussion with a third researcher.

**Statistical analysis**

Data analysis were carried out by the Stata software version 11. Standard error in each study was calculated using the binomial distribution.

The heterogeneity of reported prevalence among studies was evaluated by the Chi-square based Q test and I2 statistics (with significant level of p< 0.1). Based on the rejection of homogeneity hypothesis, the random effect model was used for estimation of pooled prevalence.

Potential publication bias was examined by Begg's test and funnel plot. Also, meta-regression was used for examining the effects of potential factors contributing to the heterogeneity in the prevalence of preterm labor.

## Results

**Figure 1:**
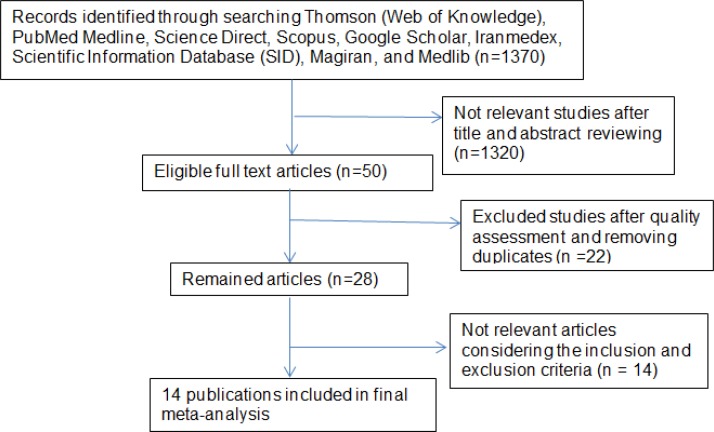
Flow diagram showing the different phases involved in searching for relevant publications

The first step of search in the mentioned databases yielded 1370 publications. In the second step, by reviewing the titles and abstracts, 50 studies were identified that were somewhat related. In the third step, after quality assessment of articles and removing duplicates, 28 studies remained. In the final step, considering the inclusion and exclusion criteria, 14 studies were selected for this meta-analysis (Table I, Figure1). The total sample size in the 14 study was 156461 people. Studies characteristics were entered in the meta-analysis are presented in table I. The highest prevalence of preterm labor was seen in Amini *et al* study in Tehran that it was equal to 30.4% (17) and the lowest prevalence was 2% in Kermanshah (18). This study was performed from 1995 to 2011. Based on the results of Begg's test, there was no evidence of publication bias (p=0.14).

**Table I:** The characteristic of studies were included in the meta-analysis of preterm labor prevalence in Iran

**Reference**	**Year**	**Study location**	**Sample size (n)**	**Prevalence (%)**
Afrakhteh M et al. (4)	1995-1999	Tehran	5628	7.23
Rajaeefard A et al. (19)	2009	Shiraz	1117	8.3
Lotf Alizadeh M et al. (20)	2002-2003	Mashhad	1979	16.4
Bayat-Mokhtary M et al. (21)	2007	Mashhad	17129	6.1
Ganji T et al. (22)	2005	Qom	1237	13.9
Khalaji Nia Z, Sadeghi Moghadam P. (23)	2007	Qom	10913	5.6
Marsusi V et al. (24)	1996	Tehran	1969	9.43
Sehati Shaghaie F et al. (25)	2009	Ardabil	3575	13.4
Sohrabi D, Ghanbari Gorkani M. (26)	2007	Zanjan	4528	7.0
Mansourghanaei M. (27)	1999-2009	Rasht	62841	5.99
Amini L et al. (17)	2009	Tehran	990	30.4
Alijahan R et al. (28)	2010-2011	Ardabil	6705	5.1
Pasdar Y et al. (18)	2010	Kermanshah	32450	2.0
Nabavizadeh SH et al. (12)	2010	Yasuj	5400	2.4

**Figure 2:**
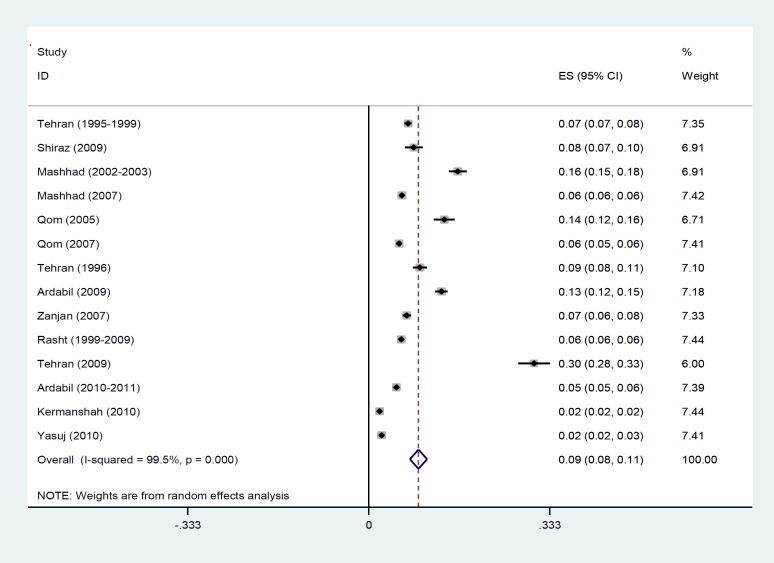
Forest plot of preterm labor prevalence in Iran using the random effect model

The results of Chi-square test based on Q test and I2 statistics showed severe heterogeneity among the reported prevalence (Q=2505. 12, p-value< 0.001 and I2= 99.5%) and consequently, the random effect model was used for the meta-analysis. The forest plot of eligible articles for estimating preterm labor prevalence in Iran is presented in figure 2. In this plot prevalence, 95% confidence interval (95% CI) and the weight assigned to each study is reported. The size of each square represents the weight of each study and the lines around it is the 95% CI. Based on the random effect model, the overall estimated prevalence of preterm in Iran was 9.2% (95% CI: 7.6 – 10.7).

Meta-regression was used to study the effects of suspected factors in heterogeneity, including sample size and year of study (Table II). The results showed that in univariate model, preterm birth was reduced with increasing in sample size, but this was not significant statistically (p=0.19).

**Figure 3.**
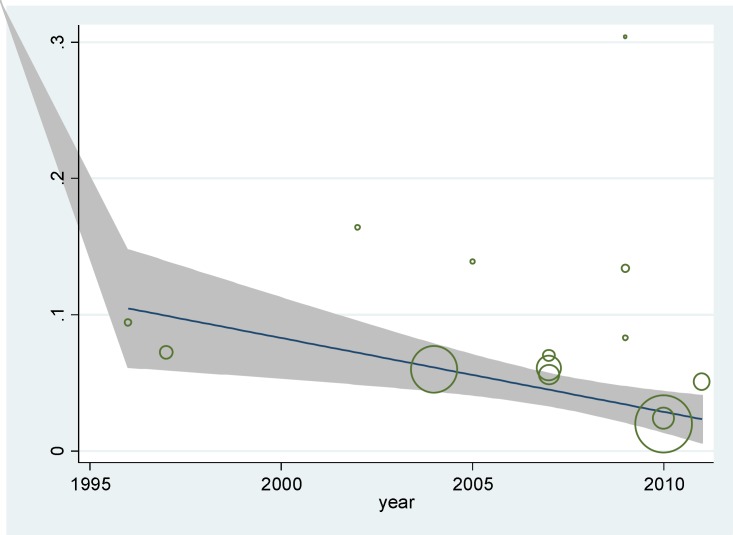
Meta-regression graph of preterm labor prevalence based on the year of study

Also as shown in Figure 3, the prevalence of preterm labor was decreased over time, but this was not statistically significant as well (p=0.76).

In the multivariate model, the variables did not have a significant effect on the heterogeneity among studies (p> 0.05).

**Table II:** The effects of suspected factors in heterogeneity

**Variable**	**Univariate**			**Multivariate**		
	**Coefficient**	**SE**	**p-value** *	**Coefficient**	**SE**	**p-value** *
**Sample size**	-1.20	1.09	0.19	-1.45	1.11	0.22
**Year of study**	-0.0013	0.004	0.76	-0.001	0.004	0.81

Note: Univariate and multivariate models meta regression

* p-value < 0.05 considered significant

## Discussion

In this study, the overall prevalence of preterm birth based on the results of 14 reviewed studies was estimated 9.2%. Since the meta-analysis use sample size of all studies, it provide*s *a better estimation of the desired indicator (14, 15), therefor this overall indicator can be used in national health planning and policymaking.

Among all of the reviewed studies, the highest prevalence of preterm labor was reported in the study of Mirzaie and Mohammah-Alizadeh among pregnant women referring to the Delivery Ward of Afzalipour Hospital in Kerman (29). Since their study was not a community based on the study and it was not according to the researchers commenting, high prevalence in this study was due to the high rate of referrals from all parts of the province to this hospital for its facilities in having neonatal intensive care unit, therefore it was excluded this study from meta-analysis.

It can be concluded that, hospitals that have a high risk deliveries, the prevalence of preterm birth may be also higher. In contrast, unlike the previous study, which was conducted by Pasdar *et al* in Kermanshah on all pregnancies during the year, the lowest preterm prevalence was found (18).

Prevalence of preterm birth is different among regions and countries. Worldwide an estimated 11.1% of all live births were born preterm in 2010 (30). Also, based on the World Health Organization 2011 report, the prevalence of preterm delivery in 184 countries was between 5 to 18%, which 60% of these deliveries occurred in Africa and South Asia. In low-income countries, on average, 12% of babies are born prematurely; in contrast, this rate is 9% in high-income countries (3). However, in some high-income countries (such as the United States with 12% and Australia with 10.9%) premature birth is also considerable (7). In this study, the prevalence of preterm delivery was estimated slightly lower than low-income countries, and was similar to the high-income countries. Also, according to the latest reports, ten countries with the highest rates of preterm birth per 100 live births in the world consists of Malawi: 18.1, Comoros: 16.7, Congo: 16.7, Zimbabwe: 16.6, Equatorial Guinea: 16.5, Mozambique: 16.4, Gabon: 16.3, Pakistan: 15.8 and Mauritania: 15.4 (5, 7). The preterm birth rate has been reported 15.7% in Pakistan and 12.8% in Jordan (31, 32).

The results of this study showed that the prevalence of preterm delivery has reduced by increasing the sample size of studies, although were not statistically significant. These findings suggest that studies with low sample size may lead to kind of sampling bias from high-risk pregnancies. Therefor the appropriate sample size and sampling should be considered in these studies.

Based on the result of the present study, the prevalence of preterm birth has decreased over the time with a gentle slope. While based on the results of the Blencowe *et al* study, preterm delivery in 184 countries from 1990 to 2010, only 3 countries had a decreasing trend, 14 countries had stable rate and all others showed an increasing trend during 20 years (7). Although the reduction in preterm birth during almost 15 years of our study was not statistically significant, but this slight reduction is probably due to the desirable reproductive health or health education programs (33, 34). The main limitation of this study was that it could not classified studies by age-groups, because of the different age distributions of subjects in eligible studies.

## Conclusion

Finally, it can be concluded that the present systematic review and meta-analysis summarized the results of previous studies and provided a comprehensive view about the preterm delivery in Iran. Although compared to the developing countries, this prevalence of preterm delivery is acceptable in Iran, but in order to achieve a more desirable level and reduce the rate in the coming years, identifying affecting factors and interventional and preventive actions seem necessary.
